# STING protects against cardiac dysfunction and remodelling by blocking autophagy

**DOI:** 10.1186/s12964-021-00793-0

**Published:** 2021-11-08

**Authors:** Rui Xiong, Ning Li, Lei Chen, Wei Wang, Bo Wang, Wenyang Jiang, Qing Geng

**Affiliations:** 1 Department of Thoracic Surgery, Renmin Hospital of Wuhan University, Wuhan, China; 2 Department of Orthopedics, Renmin Hospital of Wuhan University, Wuhan, China

**Keywords:** STING, Cardiac remodelling, Inflammation, Autophagy, ULK1

## Abstract

**Background:**

Heart failure, which is characterized by cardiac remodelling, is one of the most common chronic diseases in the aged. Stimulator of interferon genes (STING) acts as an indispensable molecule modulating immune response and inflammation in many diseases. However, the effects of STING on cardiomyopathy, especially cardiac remodelling are still largely unknown. This study was designed to investigate whether STING could affect cardiac remodelling and to explore the potential mechanisms.

**Methods:**

In vivo*,* aortic binding (AB) surgery was performed to construct the mice model of cardiac remodelling. A DNA microinjection system was used to trigger STING overexpression in mice. The STING mRNA and protein expression levels in mice heart were measured, and the cardiac hypertrophy, fibrosis, inflammation and cardiac function were also evaluated. In vitro*,* cardiomyocytes stimulated by Ang II and cardiac fibroblasts stimulated by TGF-β to performed to further study effects of STING on cardiac hypertrophy and fibroblast. In terms of mechanisms, the level of autophagy was detected in mice challenged with AB. Rapamycin, a canonical autophagy inducer, intraperitoneal injected into mice to study possible potential pathway.

**Results:**

In vivo, the STING mRNA and protein expression levels in mice heart challenged with AB for 6 weeks were significantly increased. STING overexpression significantly mitigated cardiac hypertrophy, fibrosis and inflammation, apart from improving cardiac function. In vitro*,* experiments further disclosed that STING overexpression in cardiomyocytes induced by Ang II significantly inhibited the level of cardiomyocyte cross-section area and the ANP mRNA. Meanwhile, TGF-β-induced the increase of α-SMA content and collagen synthesis in cardiac fibroblasts could be also blocked by STING overexpression. In terms of mechanisms, mice challenged with AB showed higher level of autophagy compared with the normal mice. However, STING overexpression could reverse the activation of autophagy triggered by AB. Rapamycin, a canonical autophagy inducer, offset the cardioprotective effects of STING in mice challenged with AB. Finally, further experiments unveiled that STING may inhibit autophagy by phosphorylating ULK1 on serine757.

**Conclusions:**

STING may prevent cardiac remodelling induced by pressure overload by inhibiting autophagy, which could be a promising therapeutic target in heart failure.

**Video Abstract**

**Supplementary Information:**

The online version contains supplementary material available at 10.1186/s12964-021-00793-0.

## Background

Cardiac remodelling is defined as the abnormal alterations in cardiac structure, shape, and function stimulated by pathophysiologic condition, which is implicated with a great many cardiac disorders, involving myocardial ischemia, myocardial infarction, diabetic cardiomyopathy, hypertrophic cardiomyopathy, doxorubicin-induced cardiac fibrosis as well as heart failure [1–3]. In particular, cardiac remodelling causes the increased morbidity and mortality among the elderly each year, which leads to sever economy burden globally [4]. Mountainous evidence has proved that cardiomyocyte hypertrophy and abnormal activation of cardiac fibroblast are the key mechanisms contributing to cardiac remodeling [5]. Thus, genes and proteins which could selectively slow down the progression of cardiac fibrosis and hypertrophy would be of great therapeutic interest.

Autophagy performs housekeeping functions for some degradation and recycling of cytoplasmic proteins and organelles, which plays a crucial role in adverse cardiac remodelling to regulate cellular homeostasis and cardiac function in the heart [6]. Cardiomyocyte autophagy and cardiac fibroblast autophagy are the primary sources of cardiac remodelling, but the beneficial or aggravating role of the autophagy in cardiac remodelling remains controversial for the reason that both “excessive” and “insufficient” autophagy in heart are detrimental [7]. Further study needs to be carried out to illustrate the precise regulatory mechanisms of autophagy in cardiac remodelling.

Stimulator of interferon gene (STING) is an important regulator of DNA sensing pathways. It is physiologically embedded in the endoplasmic reticulum and induces the production of type I interferon by activating nuclear factor (NF)-κB and interferons (IFN) regulator 3 (IRF3) pathways [8]. Previous study has demonstrated that in live Gram-positive bacteria, STING could sense cell viability to modulate pathogen-associated molecular patterns (PAMPs)-mediated autophagy of the endoplasmic reticulum [9]. In turn, STING could be also degraded by p62/SQSTM1-mediated autophagy [10]. A recent study showed that STING from the sea anemone *Nematostella vectensis* could trigger autophagy rather than the production of interferon when treated with cyclic GMP-AMP (cGAMP), indicating that autophagy triggered by STING is independent of IRF3 activation and may be an original function of the cGAS/STING signaling pathway [11]. Our recent study found that STING/IRF3 in cardiomyocytes could lead to sepsis-induced cardiac injury and dysfunction by activating NLRP3 inflammasomes [12]. However, the functions of STING as well as its regulatory mechanisms on autophagy in cardiac remodelling have not been well studied yet.

In this study, we examined whether STING overexpression affected cardiac hypertrophy, cardiac fibrosis, cardiac dysfunction and inflammation in the context of pressure overload. Meantime, we further elucidated the possible mechanisms of STING in cardiac remodelling.

## Materials and methods

### Reagents and antibodies

Rapamycin (≥ 99.93% purity, as determined by high-performance liquid chromatography), chloroquine (99.50% purity), Angiotensin II (99.48% purity) and TGF-β were obtained from MCE (Shanghai, China). Primary antibodies against STING (1:1000), Beclin1 (1:1000), AMPK alpha 1 + AMPK alpha 2 (1:1000), phosphorylated-AMPK alpha 1^T183^(p-AMPK alpha 1^T183^) + phosphorylated-AMPK alpha 2^T172^(p-AMPK alpha 2^T172^) (1:1000), mTOR (1:1000), phosphorylated-mTOR (p-mTOR^S2448^) (1:1000) were bought from Abcam (Cambridge, UK). Antibodies for Atg7 (1:1000), Atg12 (1:1000), LC3B (1:1000), LC3I//II (1:1000), GAPDH (1:1000), total ULK1 (1:1000), and phosphorylated-ULK1^ser757^ (p-ULK1^ser757^, 1:1000) were purchased from Cell Signaling Technology (Danvers, MA, USA). Anti-rabbit/mouse EnVisionTM+/HRP reagent for immunohistochemical staining was purchased from Gene Technology (Shanghai, China) and secondary antibody used for immunoblotting was obtained from LI-COR Biosciences.

### Animals and treatment

The cardiomyocyte-specific overexpressed STING transgenic (TG) mice, constructed by integrating the STING target gene into the genome of wild type (WT) C57/B6 mice using microinjection, were purchased from the Model Animal Research Center of Nanjing University, Nanjing, China. Male C57/B6 mice were purchased from the Institute of Laboratory Animal Science, Chinese Academy of Medical Sciences (Beijing, China). The WT mice and TG mice used in this study were 8–10 weeks old with body weight 25.3 ± 6 g. All animal experimental procedures were approved by the Animal Experimentation Ethics Committee of Wuhan University (Protocol No. 00013274) and comply with the Guide for the Care and Use of Laboratory Animals by the US National Institutes of Health (NIH Publication No. 85-23, revised in 1996).

The mice were subjected to aortic banding (AB) surgery or sham operation as described previously [13].

The mice hearts and lungs were harvested and weighed after they were sacrificed using sodium pentobarbital. Then data involving the ratios of heart weight/body weight (HW/BW, mg/g), lung weight/body weight (LW/BW, mg/g), and heart weight/tibia length (HW/TL, mg/mm) was collected.

To activate autophagy, Rapamycin (2.0 mg/kg) was used via intraperitoneal injection every other day for 4 weeks before AB surgery.

### Echocardiography

On the basis of the previous study, transthoracic echocardiography was used to collect the mean echocardiographic parameters of 3 to 5 cardiac cycles, including heart rate (BPM), ejection fraction, ejection fraction, left ventricular end-diastolic diameter (LVEDD) and left ventricular end-systolic diameter (LVESD) [13].

### Histological analysis

Simply put, the mouse heart was fixed with 4% formaldehyde overnight, then embedded in paraffin to make 5-μm sections. Next, according to our previous study, hematoxylin eosin (H&E) staining and Picrosirius Red (PSR) staining were carried out [14], aiming to observe the sectional area of cardiomyocytes and collagen volume in cardiac tissue.

Immunohistochemical staining was carried out to evaluate the content of inflammatory cells infiltration and LC3B. Endogenous peroxidase was inhibited by 3% hydrogen peroxide and the nonspecific binding of the antibody were blocked with 10% goat serum. Then, the sections were incubated with anti-CD45 (1:100), anti-CD68 (1:200), or anti LC3B (1:100) overnight at 4 °C as described previously [12].

### Cell culture and treatment

Neonatal mice cardiomyocytes as well as cardiac fibroblasts were isolated and cultured based on previous description [15, 16]. To overexpress STING, cardiomyocytes and cardiac fibroblasts were transfected with Ad-STING (MOI = 10) or adenovirus harboring no overexpression sequence (Ad-Ctrl) for 6 h. When the cells reached 75% confluence, cardiomyocytes were treated with AngII (1 μM) while cardiac fibroblasts were treated with TGF-β (10 ng/ml) for 24 h. Experiments were performed at least 3 times in duplicate. To knock down the expression of STING, cardiomyocytes and cardiac fibroblasts were incubated with STING siRNA, as well as the scrambled siRNA (designed and synthesized by Sangon Biotech, Shanghai, China). Cardiomyocytes and cardiac fibroblasts in our study were transfected using Lipofectamine 3000 (Thermo Fisher Scientific, Waltham, MA, USA) based on the manufacturer’s instructions. When the cells reached 75% confluence, Cardiomyocytes and cardiac fibroblasts were treated with Ang II or TGF-β for 24 h in the presence/absence of STING. Chloroquine (10 μM) was added 2 h before Ang II or TGF-β stimulation [17].

### Western blot and real-time PCR

Western blot was performed as our previously described^17^. Extracting the total proteins of fresh ventricle tissues or iced cell lysates and quantified, approximately 50 μg of total proteins were loaded on an SDS/PAGE gel. The resolved proteins were subsequently transferred onto polyvinylidene fluoride (PVDF) membranes. After that, membranes were put into 5% non-fat milk with PBS/0.1% Tween and blocked for 1 h. Next, membranes containing target proteins were incubated with primary antibodies overnight at 4 °C. The next day, after washing with PBS/0.1% Tween, membranes were incubated with secondary antibodies conjugated to IRDye 800CW for 50 min. Finally, images of the bands with target proteins were acquired and quantified by the Odyssey infrared imaging system (Odyssey, LI-COR, Lincoln, NE).

Real-time PCR was carried out as previously described [12]. The total RNA of the frozen ventricle tissues or iced cells was extracted by using TRIzol reagent (Invitrogen, 15596-026) and cDNA was synthesized using a Transcriptor First Strand cDNA Synthesis Kit (04896866001, Roche, USA). Quantitative real-time PCR was performed using Light Cycler 480 SYBR Green I Master Mix (Roche, 04707516001). The expression levels of target genes were uniformly normalized to GAPDH. The primers in real-time PCR are listed in Table 1.

**Table 1:** Primers in real-time PCR

Gene	Species	Forward primer	Reverse primer
IL-1β	Mouse	AATGAAGGAACGGAGGAGCC	CTCCAGCCAAGCTTCCTTGT
IL-6	Mouse	GTTGCCTTCTTGGGACTGATG	ATACTGGTCTGTTGTGGGTGGT
MCP-1	Mouse	TGGCTCAGCCAGATGCAGT	CCAGCCTACTCATTGGGATCA
HMGB1	Mouse	CCGGCAAGTTTGCACAAAGA	TTGGGAGGGCGGAGAATCAA
GAPDH	Mouse	ACTCCACTCACGGCAAATTC	TCTCCATGGTGGTGAAGACA
ANP	Mouse	ACCTGCTAGACCACCTGGAG-	CCTTGGCTGTTATCTTCGGTACCGG
Collagen1	Mouse	AGGCT TCAGTGGTT TGGATG	CACCAACAGCACCATCGTTA
Collagen3	Mouse	CCCAACCCAGAGATCCCATT	GAAGCACAGGAGCAGGTGTAGA
Fibronectin	Mouse	CGGTGGCTGTCAGTCAGA	TCCCACTGCTGATTTATC
CTGF	Mouse	TGTGTGATGAGCCCAAGGAC	AGTTGGCTCGCATCATAGTTG
STING	Mouse	ATCTATGCTAGTCGTAGTTTA	CGTAGTGCTAGTGATTAGTC
ANP	Rat	ATGGGCTCCTTCTCCATCAC	TCTTCGGTACCGGAAGCTG
BNP	Rat	TTCCTTAATCTGTCGCCGCTGG	CAGCAGCTTCTGCATCGTGGAT
Collagen1	Rat	GAGAGAGCATGACCGATGGATT	TGGACATTAGGCGCAGGAA
Collagen3	Rat	CAGACGGGAGTTTCTCCTCGGA	GACCAGGAGGACCAGCAACTCC'
GAPDH	Rat	GACATGCCGCCTGGAGAAAC	AGCCCAGGATGCCCTTTAGT

### Immunofluorescent staining

To further access cardiomyocyte hypertrophy and the transformation of cardiac fibroblast to myofibroblast, immunofluorescent staining for α-actin and α-SMA was performed according to our published article [15]. Briefly, the cardiomyocytes or cardiac fibroblasts were fixed using 4% paraformaldehyde and permeabilized using Triton X-100 (0.2%). Cardiomyocyte hypertrophy was assessed by anti-α-actinin staining while the transformation of cardiac fibroblast to myofibroblast was evaluated by anti-α-SMA staining. DAPI dye was used to visualize cell nucleus. In addition, LC3B was also measured in the two sets to evaluate autophagy level.

### Statistical analysis

All data are presented as mean ± SD and analyzed with the software SPSS 23.3. Two-way analysis of variance (ANOVA) followed by Tukey’s test for multiple comparisons while Student’s unpaired t-test was applied to compare 2 groups. *P* value < 0.05 was regarded as statistically significant.

## Results

### STING was upregulated in cardiac remodelling

**Fig. 1:**
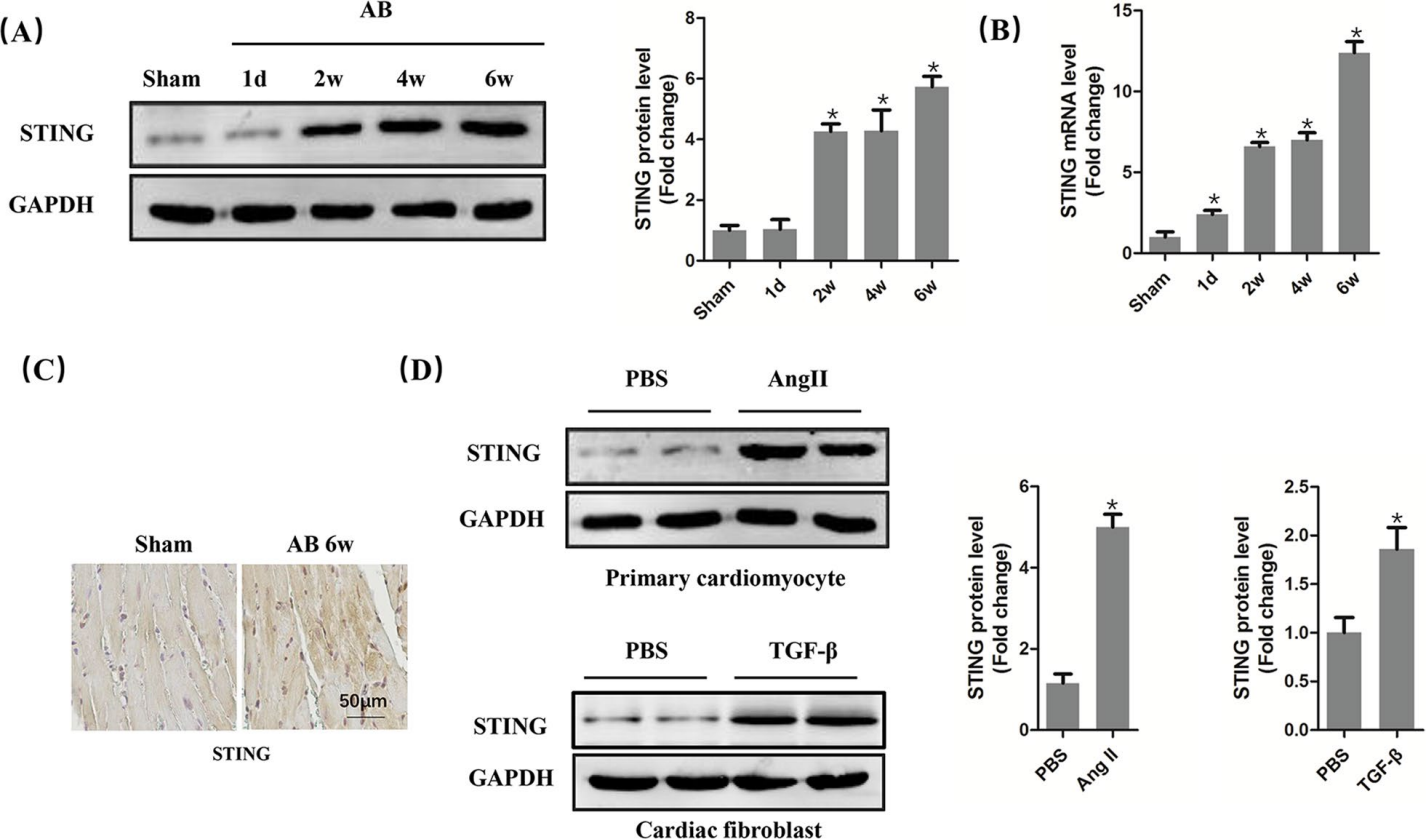
STING was upregulated in the heart in AB-induced mice, AngII-stimulated primary cardiomyocytes and TGF-β-stimulated cardiac fibroblasts. **A** Representative western blot and analysis of STING in AB-induced hearts (n = 6). **B** The mRNA analysis of STING in AB-induced hearts (n = 6). **C** Immunohistochemistry of STING in AB-induced hearts (n = 6). **D** Representative western blot and analysis of STING in AngII-stimulated primary cardiomyocytes and TGF-β-stimulated cardiac fibroblasts (n = 6). *P < 0.05 vs the Sham or PBS group

Firstly, the protein and mRNA level of STING was detected in mice heart challenged with pressure overload for different durations. The results showed the protein level of STING was significantly increased from 2 weeks for at least 6 weeks after AB surgery (Fig. 1A) while the mRNA level of STING started to increase from the first day after AB surgery (Fig. 1B). Immumohistochemical staining further proved that STING was significantly upregulated at the sixth week after AB surgery (Fig. 1C). In vitro experiment further demonstrated that the protein expression of STING significantly increased in cardiomyocytes induced by Ang II or cardiac fibroblasts induced by TGF-β (Fig. 1D).

### STING overexpression suppressed cardiac hypertrophy and fibrosis in vivo

**Fig. 2:**
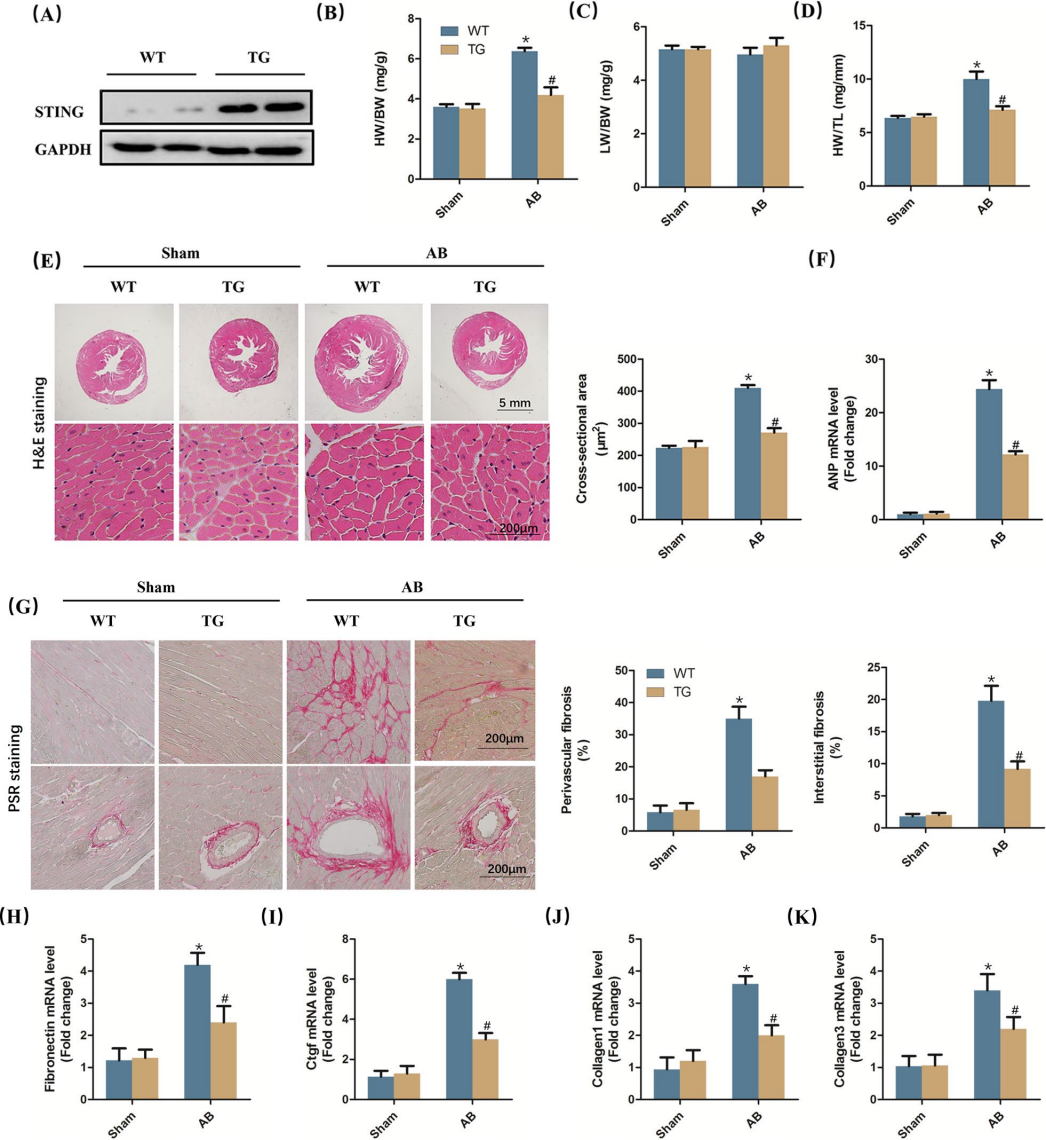
STING overexpression suppressed cardiac hypertrophy and fibrosis in vivo **A** Representative western blot of STING in wild type mice and STING TG mice (n = 6). **B**–**D** Statistical results of the heart weight (HW)/body weight (BW), lung weight (LW)/BW and HW/tibia length (TL) (n = 12). **E** Representative image of the heart with H&E staining and statistical results of cross-sectional area (n = 6). **F** The mRNA level of hypertrophic markers (ANP) (n = 6). **G** Representative image of the heart with Picrosirius Red (PSR) staining and statistical results of perivascular and interstitial fibrosis (n = 6). **H**–**K** The mRNA level of fibrotic markers (Fibronectin, Ctgf, Collagen1 and Collagen3) (n = 6), *P < 0.05 vs the Sham + WT group, ^#^*P* < 0.05 vs the AB + WT group

STING TG mice was used in this study and we used western blot to confirm that STING was overexpressed in TG mice (Fig. 2A). Subsequently, the mice were subjected to AB surgery for six weeks. As shown in Fig. 2B–D, the value of heart weight (HW)/ body weight (BW) and HW/ tibia length (TL) significantly increased in AB + WT group while STING overexpression could significantly inhibit them. It is worth noting that AB and/or STING overexpression did not affect the value of lung weight (LW)/BW in mice. Furthermore, H&E staining was used to assess the heart size as well as cross-sectional area of cardiomyocyte. As expected, STING overexpression significantly repressed the increase of heart size and cardiomyocyte size caused by pressure overload (Fig. 2E). Meanwhile, the marker of cardiac hypertrophy (ANP) could also significantly decrease in mice with AB after STING was upregulated (Fig. 2F). PSR staining was used to evaluate the condition of fibrosis in the indicated groups. As shown in Fig. 2G, the percentage of perivascular fibrosis and interstitial fibrosis was significantly enhanced in WT mice while STING TG mice displayed obviously mitigation in both perivascular fibrosis and interstitial fibrosis after AB surgery for 6 weeks. Furthermore, marks of fibrosis involving fibronectin, ctgf, collagen1 and collagen3 were detected using real-time PCR. Compared with the mice in AB + WT group, the levels of fibronectin, ctgf, collagen1 and collagen3 were significantly inhibited in AB + TG group (Fig. 2H–K).

### STING overexpression mitigated cardiac inflammatory response

**Fig. 3:**
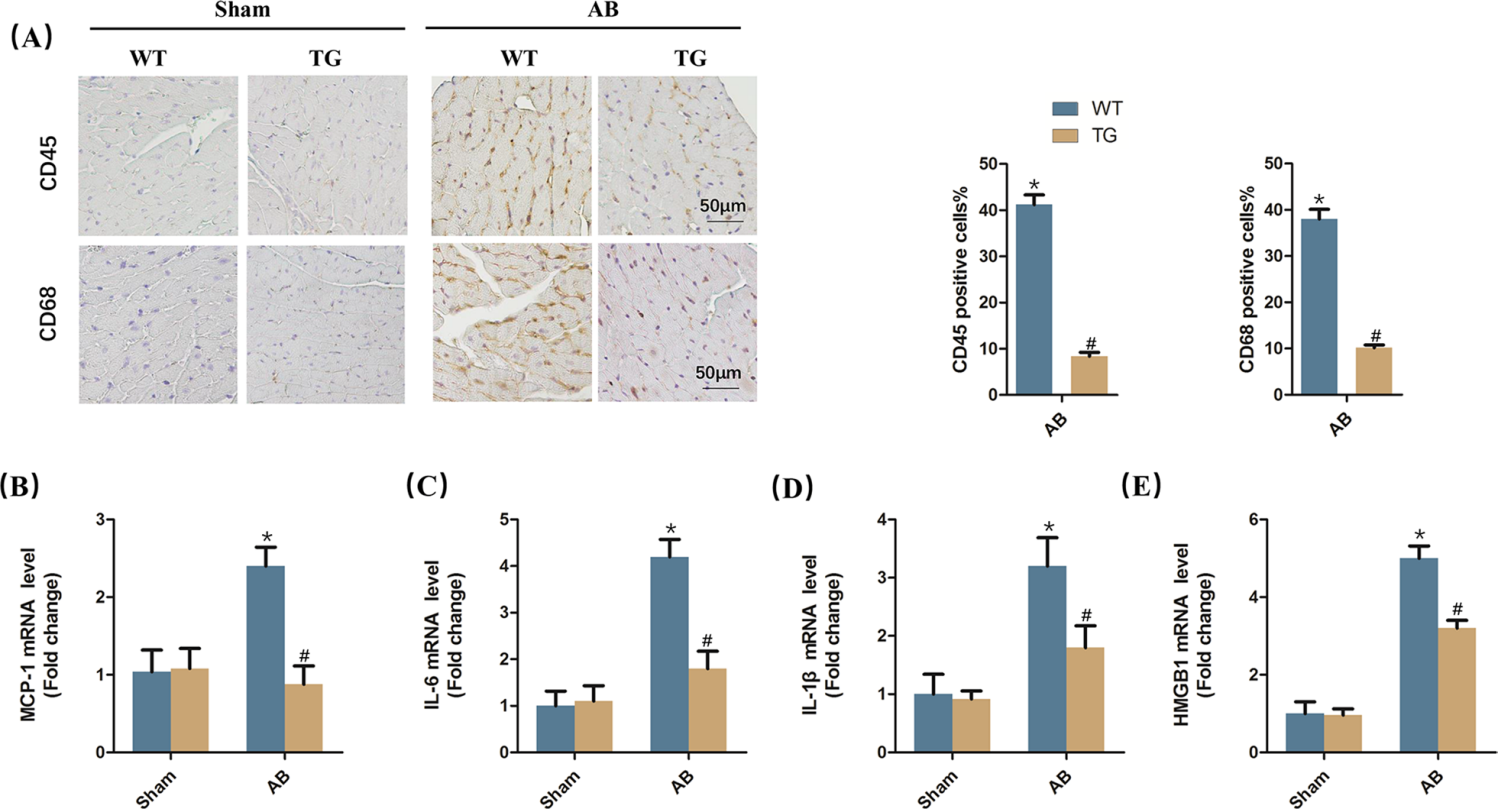
STING overexpression mitigated cardiac inflammatory response **A** Immunohistochemistry analysis of CD45 and CD68 in AB-induced hearts. Representative images and quantification (n = 6) are shown. **B**–**E** The mRNA level of proinflammatory markers (MCP-1, IL-6, IL-1β and HMGB1) (n = 6). *P < 0.05 vs the Sham + WT group, ^#^*P* < 0.05 vs the AB + WT group

Additionally, we investigated the cardiac inflammatory response in four groups. As shown in Fig. 3A, compared with WT mice challenged with AB surgery, the percentage of CD45 positive cells (leukocytes) and CD68 positive cells (macrophages) significantly reduced in TG mice challenged with AB surgery. Meanwhile, compared with AB + WT group, the mRNA levels of proinflammatory cytokines including MCP-1, IL-6, IL-1β and HMGB1 were significantly inhibited in AB + TG group (Fig. 3B–E).

### STING overexpression was involved in better cardiac function

**Fig. 4:**
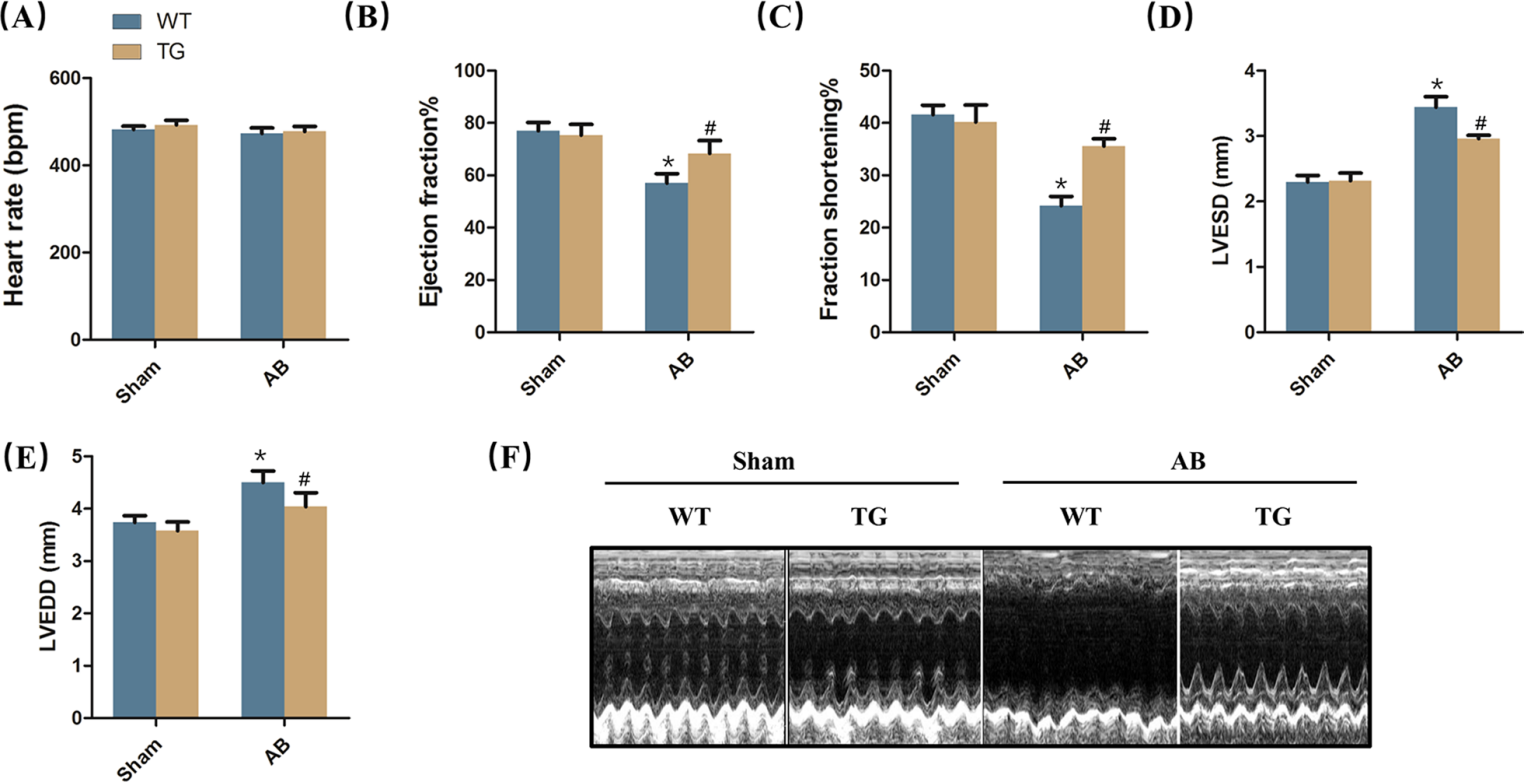
STING overexpression was involved in better cardiac function. **A**–**C** Heart rate, ejection fraction and fractional shortening of mice after AB surgery via echocardiography (n = 12). **D**–**E** Left ventricular end-diastolic diameter (LVEDD) and left ventricular end-systolic diameter (LVESD) of mice after AB surgery via echocardiography (n = 12). **F** Representative image of echocardiography. *P < 0.05 vs the Sham + WT group, ^#^*P* < 0.05 vs the AB + WT group

Next, we examined whether STING overexpression could affect cardiac function in mice subjected to AB surgery. Pressure overload and/or STING overexpression had no effects on heart rate of mice (Fig. 4A). Mice displayed deteriorative cardiac function, reflected by reduced ejection fraction and fractional shortening in response to 6-week AB surgery, which were significantly improved when STING was upregulated (Fig. 4B, C). Also, we detected the cardiac left ventricular end diameter at diastole and systole. As expected, the levels of left ventricular end-diastolic diameter (LVEDD) and left ventricular end-systolic diameter (LVESD) were significantly increased in STING TG mice after AB surgery for 6 weeks (Fig. 4D, E). The images of echocardiography also revealed that STING overexpression abolished the left ventricle enlargement induced by pressure overload (Fig. 4F).

### STING blocked cardiomyocyte hypertrophy and cardiac fibroblast activation in vitro

**Fig. 5:**
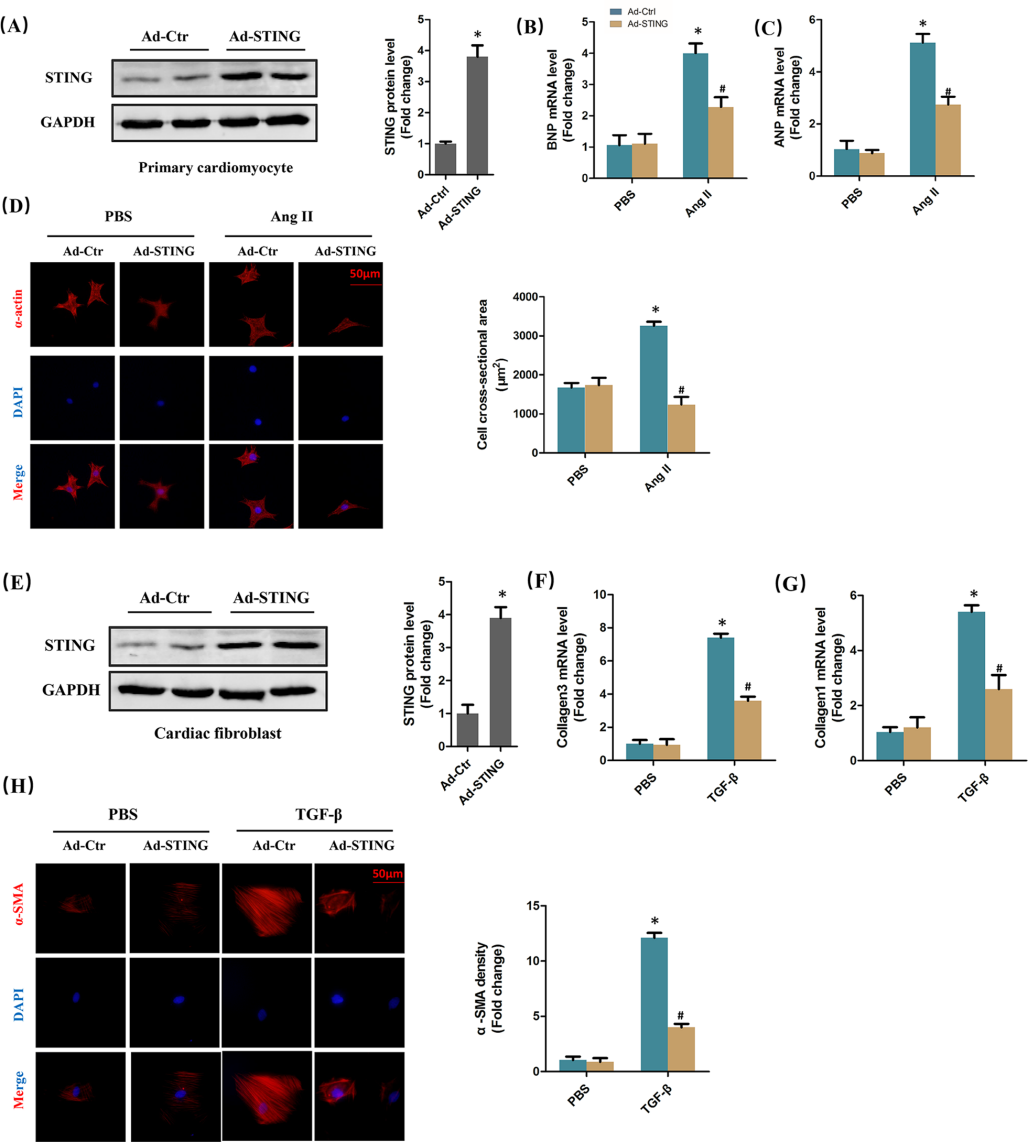
STING blocked cardiomyocyte hypertrophy and cardiac fibroblast activation in vitro*.* **A** Representative western blot of STING in primary cardiomyocytes transfected with Ad-STING (n = 6) *P < 0.05 vs Ad-Ctrl. **B**, **C** The mRNA level of hypertrophic markers (BNP and ANP) (n = 6). **D** Immunofluorescence staining and analysis of α-actin in cardiomyocytes exposed to AngII and infected with Ad-STING (n = 6) *P < 0.05 vs PBS + Ad-Ctrl group, ^#^*P* < 0.05 vs AngII + Ad-Ctrl group. **E** Representative western blot of STING in cardiac fibroblasts transfected with Ad-STING (n = 6) *P < 0.05 vs Ad-Ctrl. **F**, **G** The mRNA level of fibrotic markers (Collagen1 and Collagen3) (n = 6). **H** Immunofluorescence staining and analysis of α-SMA in cardiac fibroblasts exposed to TGF-β and infected with Ad-STING (n = 6), *P < 0.05 vs PBS + Ad-Ctrl group, ^#^*P* < 0.05 vs AngII + Ad-Ctrl group

Next, we confirmed the anti-remodelling effect of STING using adenovirus in vitro. The expression of STING was significantly enhanced in cardiomyocytes transfected with Ad-STING (Fig. 5A). STING overexpression significantly inhibited the mRNA levels of BNP as well ANP (Fig. 5B, C) and the cell cross-sectional area (Fig. 5D) of cardiomyocytes stimulated by Ang II. Similarly, the protein level of STING was significantly increased by Ad-STING in cardiac fibroblasts (Fig. 5E). Fibrotic markers involving Collagen 1, Collagen 3 as well as α-SMA were also suppressed in TGF-β-induced cardiac fibroblasts after STING was upregulated (Fig. 5F–H), indicating that STING not only inhibited collagen synthesis, but also repressed the transformation of cardiac fibroblasts to myofibroblasts.

### The cardioprotective effect of STING was mediated by autophagy inhibition

**Fig. 6:**
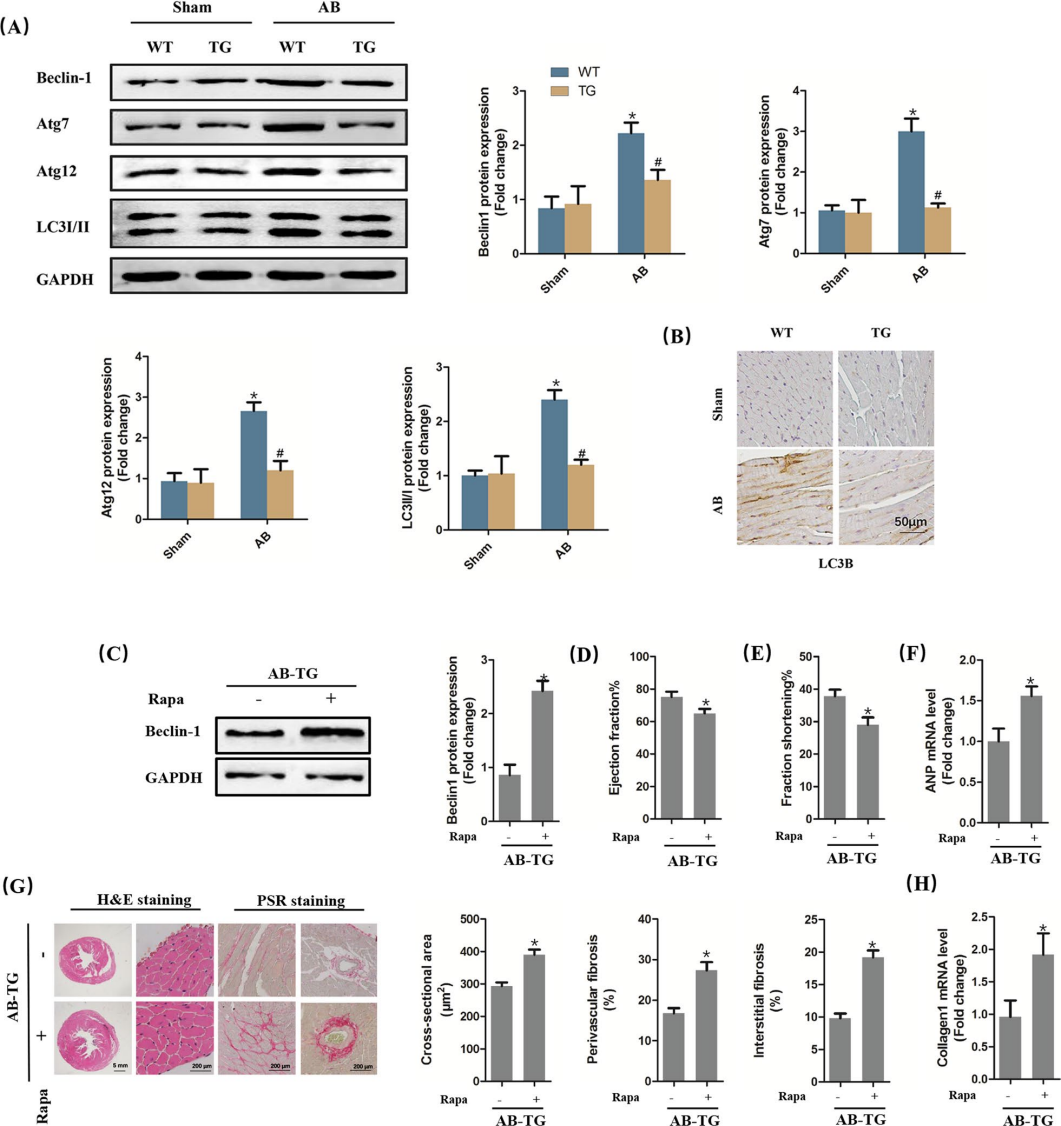
The cardioprotective effect of STING was mediated by autophagy inhibition. **A** Representative western blot and analysis of Beclin-1, Atg7, Atg12 and LC3I/II (n = 6). *P < 0.05 vs the Sham + WT group, ^#^*P* < 0.05 vs the AB + WT group. **B** Immunohistochemistry analysis of LC3B in indicated groups. **C** Representative western blot and analysis of Beclin-1 in STING TG mice with AB and rapamycin (n = 6). **D**, **E** Ejection fraction and fractional shortening in STING TG mice with AB and rapamycin via echocardiography (n = 12). **F** The mRNA level of hypertrophic markers (ANP) (n = 6). **G** Representative image of the heart with H&E staining and Picrosirius Red (PSR) staining and statistical results (n = 6). **H** The mRNA level of fibrotic markers (Collagen1) (n = 6), *P < 0.05 vs AB-TG group

Subsequently, we further explored the potential mechanism involved in the anti-remodelling effect of STING. Previous study has demonstrated that autophagy could be triggered by STING independent of IRF3 [18]. Hence, we next investigated whether STING overexpression affected myocardial autophagy activation of mice in response to pressure overload for 6 weeks. As shown in Fig. 6A, B, the protein levels of Beclin-1, Atg7, Atg12, as well as the ratio of LC3II/I were increased in mice challenged with AB surgery, which were repressed after STING was upregulated. Rapamycin is one of the potent autophagy agonists through modulating mTOR [19]. The intraperitoneal injection of Rapamycin gave rise to the increase of Beclin-1(Fig. 6C). Additionally, STING TG mice challenged with pressure overload exhibited exacerbated cardiac function when autophagy was activated (Fig. 6D, E). Meanwhile, Rapamycin also abolished the anti-hypertrophy and anti-fibrosis effects of STING in vivo (Fig. 6F–H). While in vitro, STING overexpression significantly inhibited the LC3B of cardiomyocytes stimulated by Ang II (Additional file 1: Figure 1A–B). Similarly, LC3B were also suppressed in TGF-β-induced cardiac fibroblasts after STING was upregulated (Additional file 1: Figure 1C–D). These further proved that STING protected against cardiac remodelling and dysfunction in an autophagy inhibition manner.

### STING may inhibit autophagy through phosphorylating ULK1 in heart

**Fig. 7:**
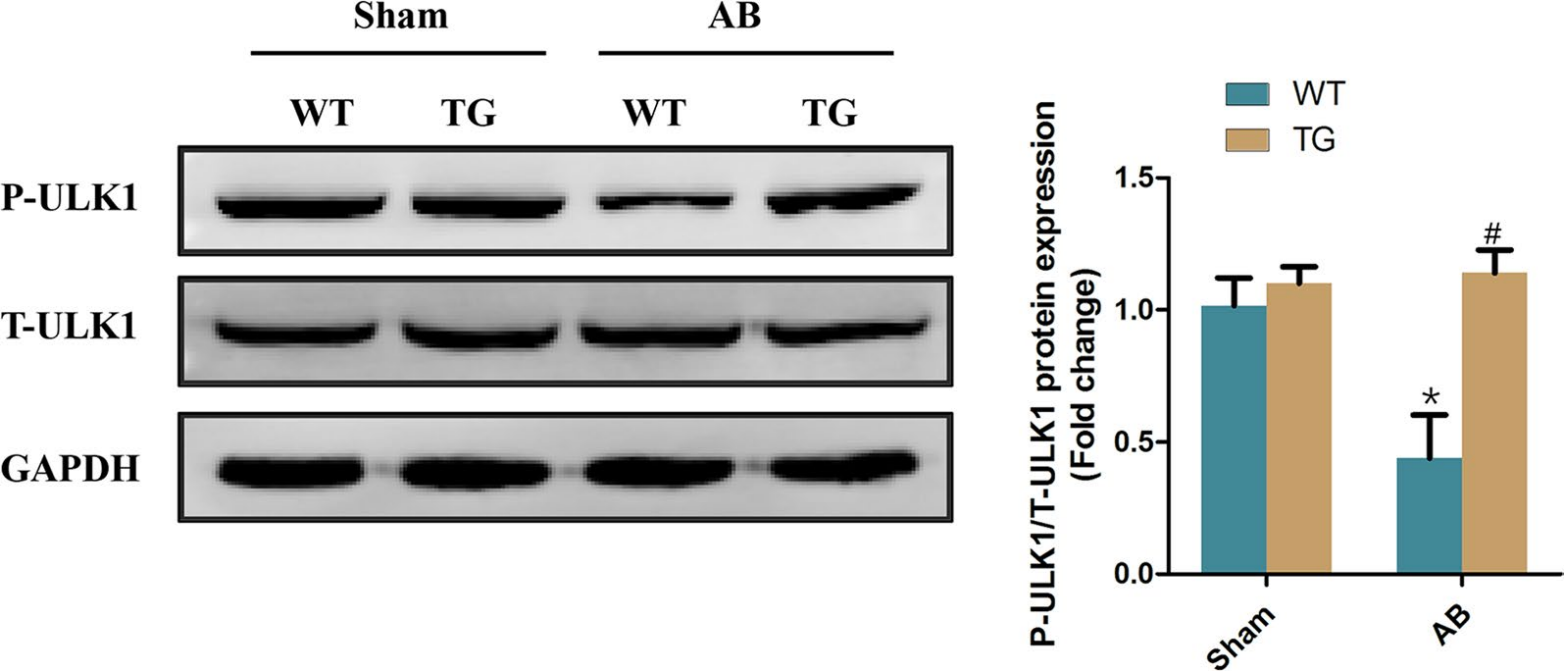
STING may inhibit autophagy through phosphorylating ULK1 in heart. Representative western blot and analysis of phosphorylated-ULK1 (P-ULK1) and total-ULK1(T-ULK1) (n = 6), *P < 0.05 vs the Sham + WT group, ^#^*P* < 0.05 vs the AB + WT group

ULK1 activation is the first step to initiate autophagy [20]. Next, we explored whether STING inhibited autophagy in heart was involved in the phosphorylation of ULK1. Western blot showed that myocardial phosphorylation level of ULK1 in mice subjected to AB surgery was significantly inhibited. However, STING significantly increased the phosphorylation level of ULK1 (Fig. 7), suggesting that ULK1 was implicated with the cardioprotective effects of STING. Considering AMPK/mTOR is the upstream signal of ULK1 [21], we further investigated whether STING promotes the phosphorylation of ULK1 by affecting the AMPK/mTOR axis. We successfully knocked down STING in cardiomyocytes (Additional file 1: Figure 2A–B). The results showed that chloroquine, a lysosomal inhibitor, inhibited the phosphorylation of AMPK, promoted the phosphorylation of mTOR and ULK1 in cardiomyocytes with Ang II stimulation. After STING was knocked down, the phosphorylation of ULK1 decreased significantly, but the phosphorylation of AMPK and mTOR were not affected (Additional file 1: Figure 2C–F). We further successfully knocked down STING in cardiac fibroblasts (Additional file 1: Fig. 2G–H). The results presented a similar conclusion with cardiomyocytes (Additional file 1: Fig. 2I–L). These indicating that STING phosphorylates ULK1 in an AMPK/mTOR-independent manner.

## Discussion

Our study disclosed the cardioprotective potential of STING in pressure overload-induced cardiac remodelling, using a realistic model of human heart failure. In detail, STING could participate in cardioprotection from the following aspects: (1) reduction of cardiac fibrosis and cardiac hypertrophy; (2) suppression of proinfammatory cytokines production; (3) improvement of cardiac function; (4) inhibition of autophagy; (5) phosphorylation of ULK1.

As a conserved, homeostatic process for the degradation and recycle of intracellular damaged organelles and macromolecules, autophagy has been proved to exert essential roles in improve cardiac function with cardiac remodeling [22]. In fact, the role of autophagy in cardiac hyperthrophy and heart failure is still controversial. Cardiac-specific Atg5 deficiency mice exhibited worse cardiac function and more serious cardiac hypertrophy after pressure overload for 1 week compared with wild type mice [23]. Meanwhile, Atg5 null mice displayed significantly less suppression of left ventricle hypertrophy than the control mice after 7 days, unloading [24]. One clinical research also demonstrated that myofilament alterations and autophagic vacuoles was associated with better prognosis in patients with dilated cardiomyopathy, indicating that autophagy activation may serve as an adaptive response [25]. On the contrary, Beclin-1 overexpression or some pharmacological agonists aggravated cardiac hypertrophy by activating autophagy [6, 26], which implied that excessive autophagy may give cell death and act as an undesirable role in maintain cardiac function. Accumulating evidence unveiled that both excessive and insufficient autophagy could trigger cardiac hypertrophy for the reason that the dynamic balance between cardiac protein degradation and synthesis determines myocardial mass [6]. As for cardiac fibrosis, the maladaptive effect of autophagy is relatively defined. Studies have shown that TGF-β could promote autophagy in human tubular epithelial kidney cells and mouse fibroblasts [27, 28]. In human atrial myofibroblasts, TGF-β may simultaneously trigger autophagy and fibrosis in human atrial myofibroblasts and pharmacological inhibition of autophagy caused the decrease in TGF-β-induced cardiac fibrosis [29]. In our study, autophagy was activated in mice subjected to pressure overload, which was accompanied with deteriorated hypertrophic growth, worse cardiac function, increased fibrosis as well as inflammatory response. However, autophagy inhibition significantly repressed pathological alteration of heart, hinting that autophagy activation may play a negative role in the pathogenesis of chronic heart failure.

STING protein is located on the endoplasmic reticulum, which was firstly discovered in 2008. The original function of STING is to promote type I IFN production upon the stimulation cyclic dinucleotides. In recent years, STING has been recognized as a critical and promising target for various cardiovascular diseases. For instance, STING-IFRF3 axis is critically associated in obesity–induced endothelial injury and inflammation, the inhibition of which could partially protected against adipose tissue inflammation, insulin resistance, obesity, as well as glucose intolerance [30]. In the model of myocardial infarction, suppression of IRF3-dependent signaling leaded to reduced levels of cardiac inflammatory cytokines and decreased inflammatory cell infiltration, apart from relieving left ventricular dilation and improving cardiac function [31]. Our lab previously reported that STING-IRF3 axis could be initiated by lipopolysaccharide, thus trigger cardiomyocytes apoptosis and pyroptosis by activating NLRP3 inflammasome [12]. In the present study, STING overexpression significantly improved cardiac function, and alleviated AB-induced cardiac hypertrophy, fibrosis as well as inflammation. The abnormal protein synthesis within the endoplasmic reticulum could lead to the accumulation of unfolded proteins, thereby giving rise to endoplasmic reticulum stress as well as the unfolded protein response. Endoplasmic reticulum stress has been identified one of the main mechanisms contributing to cardiac remodeling [32]. In hepatocytes, endoplasmic reticulum stress could activate IRF3 via STING, eventually causing hepatocyte apoptosis [8]. Clinical study has disclosed that endoplasmic reticulum stress is activated during cardiac remodelling, which exerts a crucial role in maintaining cell homeostasis [8]. Endoplasmic reticulum stress is also linked to the autophagic response and the endoplasmic reticulum stress-autophagy pathway is involved in pathological cardiac hypertrophy [33]. Based on these studies, we speculate that STING may serve as a bridge between endoplasmic reticulum stress and autophagy.

Apoptotic signaling-dependent disruption of ULK1, a pro-autophagic protein negatively regulating STING, could stimulate STING-dependent IRF3 activation by phosphorylating STING at serine 366 site [34, 35], indicating that STING could directly interact with ULK1. On the other hand, mTOR can phosphorylate ULK1 at its serine 757 site and inhibit its kinase activity, thus blocking autophagy [36]. Considering that AMPK/mTOR/ULK1 axis is deeply involved in autophagy process [21], we further investigated whether STING promotes the phosphorylation of ULK1 at its serine 757 site by affecting the AMPK / mTOR axis. After STING was knocked down in cardiomyocytes or cardiac fibroblasts, the phosphorylation of ULK1 decreased significantly, but the phosphorylation of AMPK and mTOR were not affected in Ang II or TGF-β + chloroquine group. These indicating that STING phosphorylates ULK1 in an AMPK / mTOR-independent manner.

It is worth noting that a relatively recent publication by Zhang et al. [37] identifies STING as a critical mediator of cardiac remodelling and ER stress, where its suppression was found to be associated with improved outcomes. Some reasons may explain the controversy. Firstly, in our study, we proved the protective effects of STING overexpression on cardiac function in cardiomyocyte-specific overexpressed STING mice, which indicated from the side that low expression or deletion of STING in cardiomyocytes would have adverse effects on cardiac function. Wang et al. also disclosed that STING suppression were harmful to cardiac function [38]. While in Zhang et al.'s study, STING conventional knockout mice were used in TAC model. We think that the loss of STING in cardiomyocytes is indeed detrimental to cardiac function, but the loss of STING in other tissues and cells may have a series of direct or indirect protective effects on the heart, and such protective effects are greater than the adverse factors. Therefore, their research showed that STING suppression had protective effects on cardiac function, which is the opposite of what we found. Maybe further experiments are needed to explain this phenomenon. More importantly, STING as an important element to activate innate immunity, plays an important role in immune cells, especially in macrophages [39, 40], so it is actually difficult to demonstrate the value of STING in cardiomyocytes in conventional knockout mice. Finally, the mice were subjected to AB surgery for six weeks in our study, but only four weeks for their mice. Different modeling time may also cause inconsistencies in experimental results.

## Conclusions

In summary, we demonstrate that STING, an endoplasmic reticulum adaptor, may ameliorate the development of AB-induced cardiac fibrosis and hypertrophy by repressing autophagy. STING can increase the phosphorylation of ULK1, thus decreasing inflammation and autophagy in heart.

## Supplementary Information

**Additional file 1.** STING overexpression inhibited LC3B expression in vitro and STING phosphorylates ULK1 in an AMPK/mTOR-independent manner.

## Data Availability

The datasets used and/or analysed during the current study are available from the corresponding author on reasonable request.

## Abbreviations

STING: Stimulator of interferon genes
AB: Aortic binding
NF-κB: Nuclear factor (NF)-κB
IRF3: Interferons (IFN) regulator 3
PAMPs: Pathogen-associated molecular patterns
cGAMP: Cyclic GMP-AMP
p-ULK1ser757: Phosphorylated-ULK1ser757
WT: Wild type
TG: Transgenic
HW/BW: Heart weight/body weight
LW/BW: Lung weight/body weight
HW/TL: Heart weight/tibia length
LVEDD: Left ventricular end-diastolic diameter
LVESD: Left ventricular end-systolic diameter
H&E: Hematoxylin eosin
PSR: Picrosirius Red
