# Supplementary material for: STING protects against cardiac dysfunction and remodelling by blocking autophagy
Source: Cell Commun Signal. 2021 Nov 8;19:109. doi: 10.1186/s12964-021-00793-0 (PMC8576910; doi:10.1186/s12964-021-00793-0)

**Supplement Data**

**Figure 1.** STING overexpression inhibited LC3B expression *in vitro*.

(A-B) Immunofluorescence staining and analysis of LC3B in cardiomyocytes exposed to Ang II and infected with Ad-STING (×200, n=3). (C-D) Immunofluorescence staining and analysis of LC3B in cardiac fibroblasts exposed to TGF-β and infected with Ad-STING (×200, n=3). *P < 0.05, #P>0.05


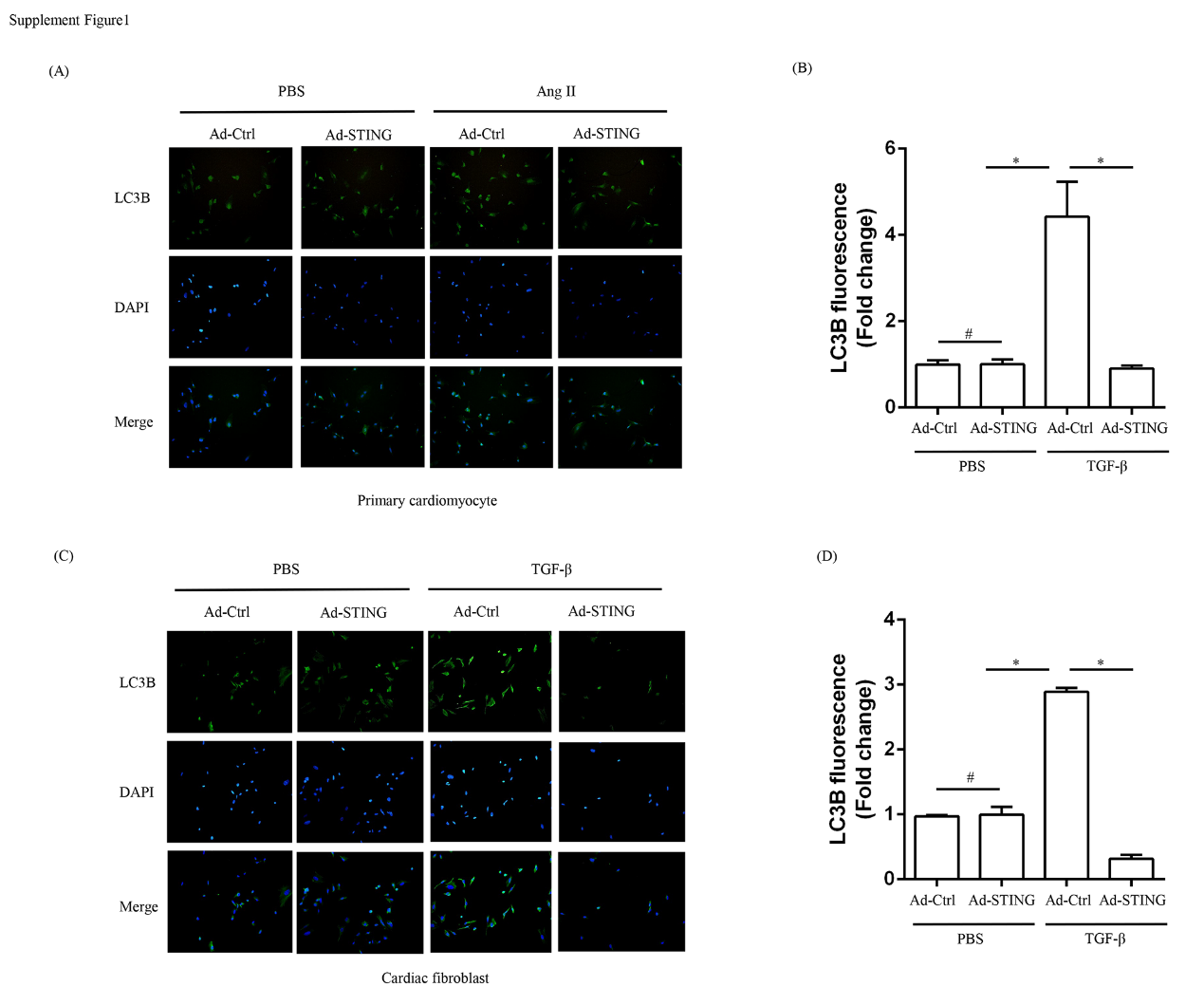


**Figure 2.** STING phosphorylates ULK1 in an AMPK / mTOR-independent manner.

(A-B) Western blots and statistical results for STING after STING was knocked down in cardiomyocytes (n=3). (C-F) Western blots and statistical results for AMPK, p-AMPK, mTOR, p-mTOR, ULK1, p-ULK1 in cardiomyocytes treated with Ang II in the presence/absence of STING(n=3). Chloroquine(10μM) was added 2h before Ang II stimulation. (G-H) Western blots and statistical results for STING after STING was knocked down in cardiac fibroblasts (n=3). (I-L) Western blots and statistical results for AMPK, p-AMPK, mTOR, p-mTOR, ULK1, p-ULK1 in cardiac fibroblasts treated with TGF-β in the presence/absence of STING(n=3). Chloroquine(10μM) was added 2h before TGF-β stimulation. *P < 0.05, #P>0.05.


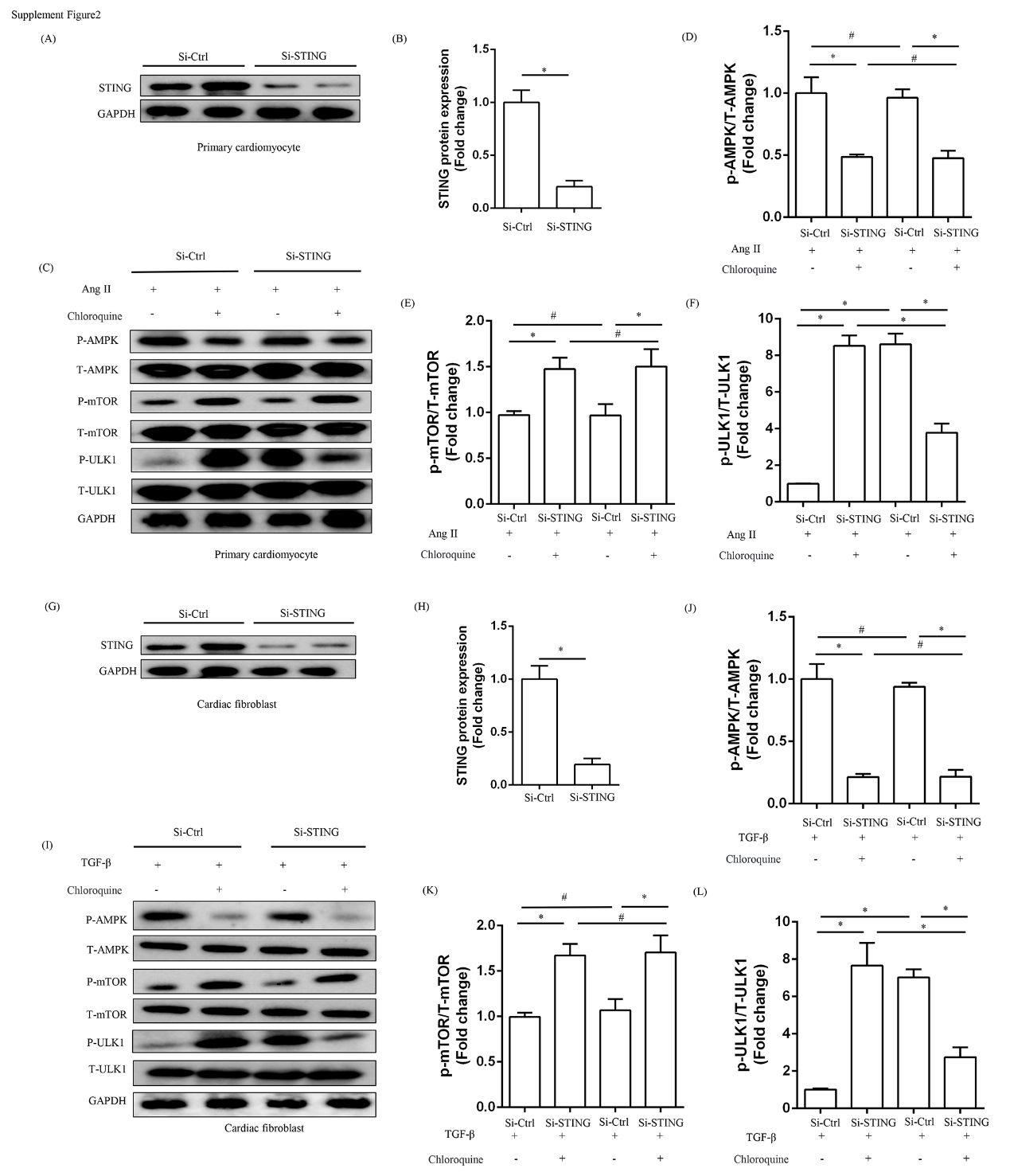

Supplement: Supplementary file 2 — Additional file 1. STING overexpression inhibited LC3B expression in vitro and STING phosphorylates ULK1 in an AMPK/mTOR-independent manner. [file 12964_2021_793_MOESM2_ESM.docx]
